# Inner Synovial Membrane Footprint of the Anterior Elbow Capsule: An Arthroscopic Boundary

**DOI:** 10.1155/2015/426974

**Published:** 2015-08-25

**Authors:** Srinath Kamineni, Abdo Bachoura, Koichi Sasaki, Danielle Reilly, Kate N. Harris, Anthony Sinai, Andrew Deane

**Affiliations:** Elbow Shoulder Research Centre (ESRC), Department of Orthopaedic Surgery and Sports Medicine, University of Kentucky, 740 South Limestone Street, K-412 Kentucky Clinic, Lexington, KY 40536-0284, USA

## Abstract

*Introduction*. The purpose of this study is to describe the inner synovial membrane (SM) of the anterior elbow capsule, both qualitatively and quantitatively. *Materials and Methods*. Twenty-two cadaveric human elbows were dissected and the distal humerus and SM attachments were digitized using a digitizer. The transepicondylar line (TEL) was used as the primary descriptor of various landmarks. The distance between the medial epicondyle and medial SM edge, SM apex overlying the coronoid fossa, the central SM nadir, and the apex of the SM insertion overlying the radial fossa and distance from the lateral epicondyle to lateral SM edge along the TEL were measured and further analyzed. Gender and side-to-side statistical comparisons were calculated. *Results*. The mean age of the subjects was 80.4 years, with six male and five female cadavers. The SM had a distinctive double arched attachment overlying the radial and coronoid fossae. No gender-based or side-to-side quantitative differences were noted. In 18 out of 22 specimens (81.8%), an infolding extension of the SM was observed overlying the medial aspect of the trochlea. The SM did not coincide with the outer fibrous attachment in any specimen. *Conclusion*. The humeral footprint of the synovial membrane of the anterior elbow capsule is more complex and not as capacious as commonly understood from the current literature. The synovial membrane nadir between the two anterior fossae may help to explain and hence preempt technical difficulties, a reduction in working arthroscopic volume in inflammatory and posttraumatic pathologies. This knowledge should allow the surgeon to approach this aspect of the anterior elbow compartment space with the confidence that detachment of this synovial attachment, to create working space, does not equate to breaching the capsule. Alternatively, stripping the synovial attachment from the anterior humerus does not constitute an anterior capsular release.

## 1. Introduction

**Figure 1:**
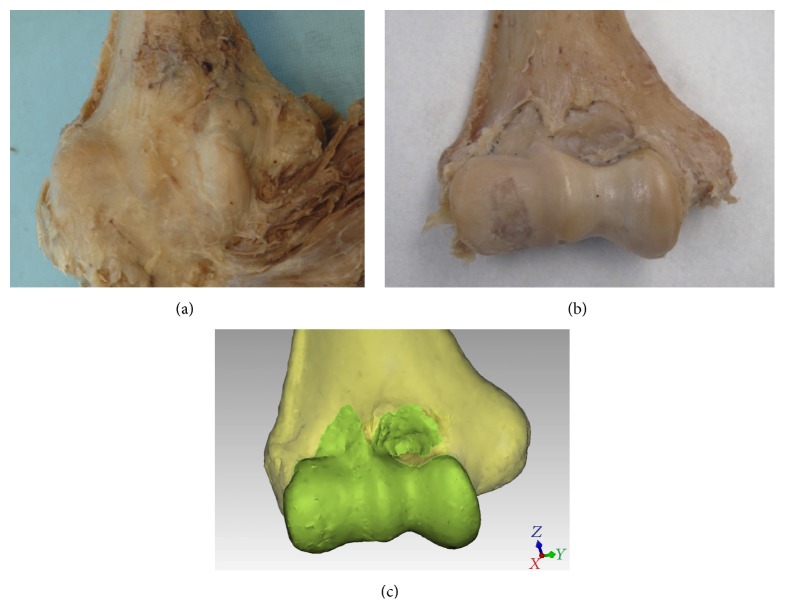
(a) Distal humerus with the anterior fibrous elbow capsule intact and the brachialis inserted on to the capsule; (b) the same specimen dissected down to the insertion of the synovial membrane; (c) digitization of the synovial membrane's insertion.

The elbow joint is bounded by a thin capsule, consistent of a broad outer fibrous capsule and an inner synovial lining [1] (Figures 1(a) and 1(b)). Often, both layers are referred to as the “joint capsule.” The articulating surfaces of the elbow, the ulnotrochlear, radiocapitellar, and radioulnar articulations are enclosed within the synovial membrane. The joint capsule plays an important role in both the normal and pathologic processes of the elbow. In the normal state, the elbow capsule imparts stability to the joint by acting as a static stabilizer [2] and provides an attachment site for the brachialis muscle, which acts as a dynamic stabilizer of the elbow [3]. The synovial membrane produces and constrains synovial fluid, vital for nourishing and lubricating the articular surfaces.

The elbow capsule bounds the three-dimensional space, which defines the working volume of the joint, an important aspect of joint anatomy, directly applicable to arthroscopy. Elbow joint arthroscopy has increasingly become a useful tool for diagnosing and treating various elbow pathologies and the indications for arthroscopy are increasing with more experience [4]. Despite the clinical importance of the elbow capsule and the relatively high complication rates of elbow arthroscopy [5], detailed morphometric studies are currently scarce [6]. In part, this has been due to the difficulty and complexity of accurately and objectively mapping and measuring the attachments of the capsule. Arthroscopy of the elbow joint necessitates accurate penetration of the capsule and navigation of the articular space within the boundaries of the inner synovial lining of the capsule. Surgeons performing open surgery of the elbow may also benefit from a detailed description of the capsule, especially during pertinent surgical procedures, such as flexion contracture release [7].

Therefore, in this study we set out to quantitatively and qualitatively describe the three-dimensional anatomic characteristics of the anterior elbow capsule or more specifically the attachments of the inner synovial membrane (SM) on the anterior surface of the distal humerus. A detailed understanding of the anatomy may be useful for open and arthroscopic surgical procedures and may potentially shed light on elbow diseases where the capsule is involved in the pathogenesis of the disorder.

## 2. Materials and Methods

A convenience sample consisting of twenty-two soft-preserved human elbows from eleven cadavers was acquired. Diligent dissection of the elbow and preservation of the inner attachments of the elbow capsule was conducted. All soft tissue other than the capsule was removed. Each humerus was then rigidly fixed in a custom made jig. The distal humerus and the shape of the inner capsular insertions were digitized with a digitizing arm (FARO, Lake Mary, FL) mounted with a 2 mm touch ball probe (Renishaw, Gloucestershire, UK). According to the manufacturer (FARO), the reported accuracy of the digitizing stylus was ±0.029 mm. Three-dimensional (3D) inspection computer software was used to register and analyze the data (Qualify version 12, Geomagic, Research Triangle Park, NC) (Figure 1).

**Figure 2:**
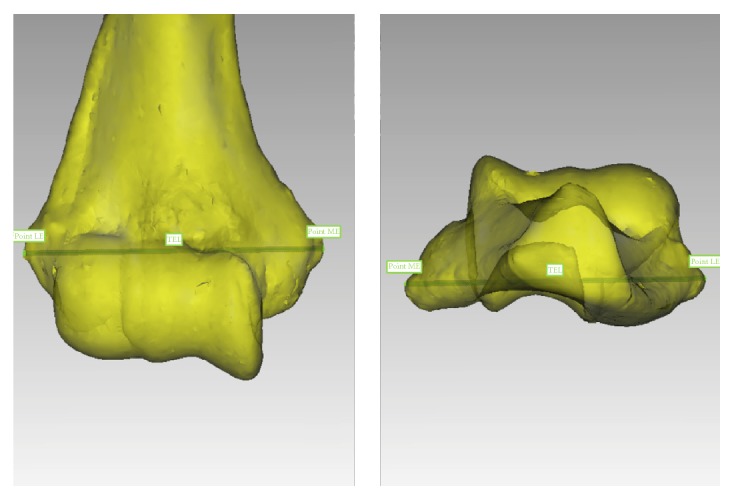
The transepicondylar line (TEL) was created by connecting the most extreme points on the medial epicondyle (ME) and lateral epicondyle (LE).

**Figure 3:**
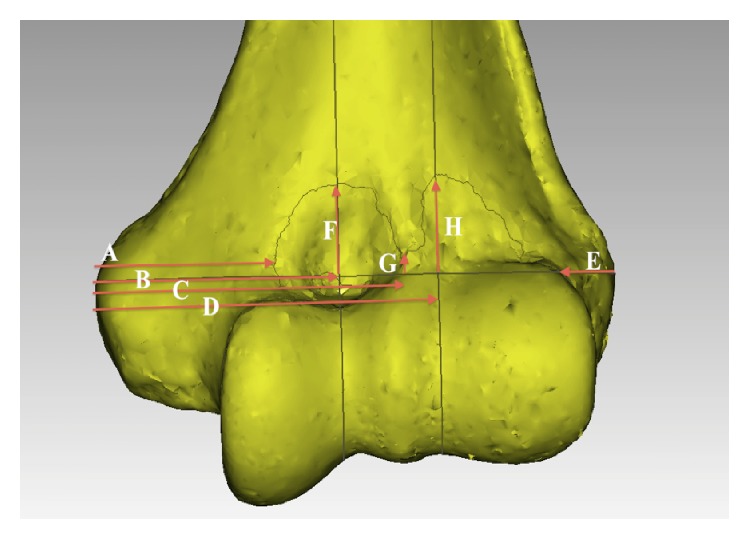
The dimensions measured were relative to the transepicondylar line (TEL). A: Horizontal distance along the TEL between the medial epicondyle (ME) and the medial edge of the synovial membrane (SM); B: horizontal distance between the ME to the apex of the SM overlying the coronoid fossa in the plane perpendicular to the TEL; C: horizontal distance between the ME and the nadir of the SM's insertion in the plane perpendicular to the TEL; D: horizontal distance between the ME to the apex of the SM overlying the radial fossa in the plane perpendicular to the TEL; E: horizontal distance along the TEL between the lateral epicondyle (LE) and the lateral edge of the SM; F: the vertical height from the TEL to the apex of the SM overlying the coronoid fossa in the plane perpendicular to the TEL; G: the vertical height from the TEL to the nadir of the SM overlying the lateral edge of the trochlea in the plane perpendicular to the TEL; H: the vertical height from the TEL to the apex of the SM overlying the radial fossa in the plane perpendicular to the TEL.

**Figure 4:**
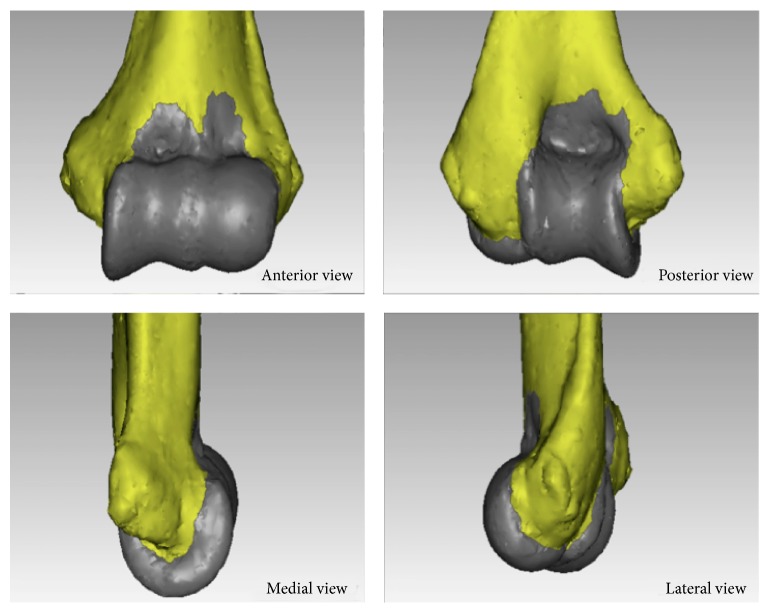
The shape of the synovial membrane's insertion and the total surface area of the distal humerus encompassed within the inner lining of the capsule.

**Figure 5:**
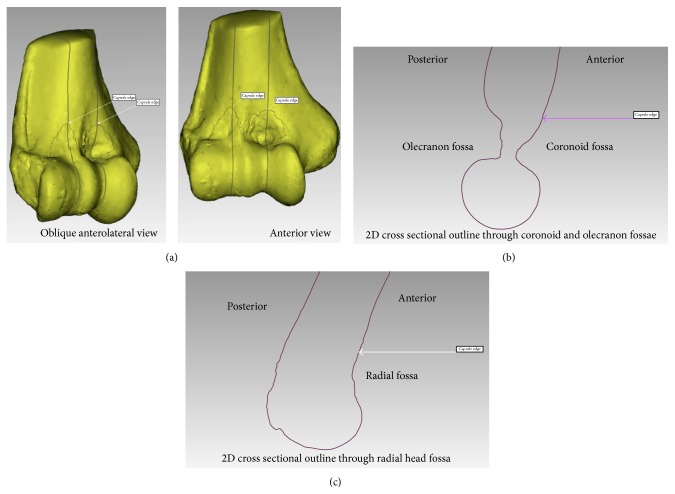
The outline of the synovial membrane's (SM) insertion on the 3D image (a) and the corresponding two-dimensional cross sectional cuts at the apex of the SM's attachment overlying the coronoid (b) and radial fossae (c); the SM attachment is marked “capsule edge.”

The most medial and lateral points on the medial and lateral epicondyles, respectively, were identified in the axial and coronal planes and marked. These points were then linked to create a transepicondylar line (TEL), which was subsequently used as the primary reference feature to describe different landmarks of the anterior capsule (Figure 2). The horizontal distance along the TEL between the medial epicondyle and the medial edge of the SM, apex of the SM overlying the coronoid fossa, the central SM nadir, and the apex of the SM insertion overlying the radial fossa was measured in millimeters and expressed as a proportion of the total length of the TEL. The distance from the lateral epicondyle to the lateral edge of the SM was measured along the TEL in the anteroposterior plane (Figure 3). The vertical height from the TEL to the apices of SM's insertions overlying the coronoid and radial fossae as well as the nadir in between the fossae was measured in a plane perpendicular to the TEL (Figure 3). The total surface area of the distal humerus encompassed within the inner lining of the capsule was also measured (Figure 4). Two-dimensional sagittal cross sections were constructed along the planes perpendicular to the TEL at the apices of the coronoid and radial fossae and the locations of the SM's attachments marked (Figure 5), to offer a better understanding of the capsular attachment relative to the anterior elbow fossae.

A Mann-Whitney* U *test was used to compare two groups. The statistics package SPSS version 20 was used (IBM Corporation, Somers, NY). Differences that had less than 0.05 probability of occurring from chance were considered statistically significant.

## 3. Results

The cadavers had a mean age of 80.4 years (range 57 to 81). There were six male cadavers and five females. In total there were twenty-two distal humeri: eleven right sided and eleven left sided.

### 3.1. Qualitative Results

**Figure 6:**
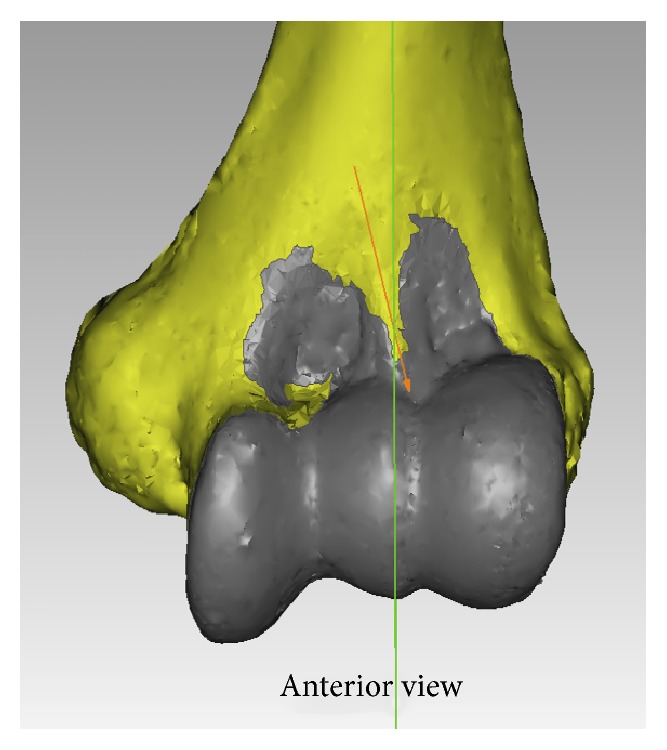
The axis of the anterior synovial membrane's insertion was rotated medially in 81.8% of cases. The arches were clearly observed to have a valgus axis relative to the axis perpendicular to the transepicondylar line, which is a close approximation of the humeral shaft axis.

**Figure 7:**
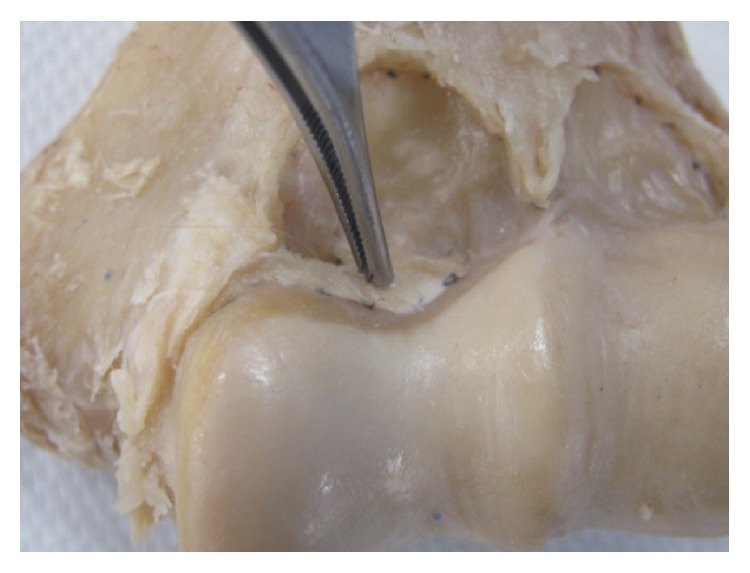
Medial extension of the synovial membrane's insertion overlying the trochlea was present in 81.8% of the specimens. This is an anterior view.

A summary of the qualitative results is presented in Table 1. The SM inserts on a bony segment anteriorly and posteriorly and wraps around the trochlea medially and the capitellum laterally, in one continuous lining (Figure 4). In all the specimens, the anterior capsule's SM insertion had a double arched structure encompassing the coronoid and radial fossae. The nadir of the arches was at the lateral end of the trochlea in all cases. In 18 (81.8%) cases, the arches were clearly observed to have a medially rotated axis or in valgus relative to the axis perpendicular to the TEL (Figure 6). In 18 out of 22 specimens (81.8%), an infolding extension of the SM was observed overlying the medial aspect of the trochlea (Figure 7). The apex of the SM's insertion overlying the coronoid fossa attached to a relatively flat or a mildly convex portion of the distal humeral surface in 18/22 specimens is shown in Figure 5(b). The capsule was inserted on the curvature of the coronoid fossa in two specimens, while two more specimens had a bony anomaly in the coronoid fossa that distorted the true location of the capsular insertion. With regard to the apex of the SM's insertion overlying the radial fossa, the insertion was on a relatively flat portion of the distal humeral surface in 21/22 (95.5%) of specimens (Figure 5(c)).

### 3.2. Quantitative Results

The quantitative results are tabulated in Tables 2–6. Males had a longer mean TEL, 70.9 mm as compared to 61.9 mm in females, *P* = 0.000 (Table 2). Subsequently, statistically significant gender differences were found for the horizontal distances between the ME and the apex of the SM overlying the coronoid fossa, ME and the nadir, and ME and the apex of the SM overlying the radial fossa (Table 3). When these measurements were considered as a proportion of the TEL, no statistical differences were found (Tables 3 and 4). No side-to-side differences for any of the measurements were found. The apex of the SM overlying the coronoid fossa was found to be 31.3 mm or 46.9% of the total length of the TEL, lateral to the ME. The apex of the SM overlying the radial fossa was found to be 44.9 mm or 67.3% of the total length of the TEL, lateral to the ME. The nadir of the SM overlying the lateral edge of the trochlea was found to be 40.5 mm or 60.7% of the total length of the TEL, lateral to the ME (Table 3). When using the TEL as a reference, the arch overlying the radial fossa was on average taller than the arch overlying the coronoid fossa (Table 4). The medial edge of the SM was found to be 23.2 mm (34.8%) of the total length of the TEL, lateral to the ME, while the lateral edge of the SM was found to be 6.5 mm (9.8%) of the total length of the TEL, medial to the LE (Table 5). The total surface area encompassed by the continuous anterior and posterior SM included the cartilaginous articular surface and noncartilaginous surfaces such as the radial and coronoid fossae. The mean area for males was 42.2 cm^2^ as compared to 33.6 cm^2^ for females, *P* = 0.000 (Table 6).

## 4. Discussion

The elbow capsule plays an important role in both normal and abnormal elbow function and has previously been identified as a potential source of pathological perturbation of elbow function [8–10]. Diseases of the anterior and posterior capsules can present independently of each other and their treatment necessitates different surgical and arthroscopic approaches [11]. The focus of this study therefore was to describe the morphology of the synovial lining of the anterior elbow capsule. A detailed morphometric understanding of the anterior capsule is of benefit to physicians who treat elbow disease. Previous anatomic studies of the elbow capsule have focused on the relations between the adjacent neurovascular structures and the outer fibrous capsular layer of the elbow joint [6, 12], but we have studied the morphometry of the inner synovial membrane of the joint capsule because this directly constrains the volumetric space of the joint.

The distal humerus has a complex bony and articular anatomy that makes accurate, objective, and reproducible studies of its morphology and its surrounding soft tissues challenging. Using computer software, the transepicondylar line was used as a normalizing element of the elbow and a reference feature to measure subsequent dimensions. In the current study, the most evident and consistent qualitative finding was the double arched shape of the synovial membrane (100% of specimens) (Figures 1(a) and 1(b)). Although this shape has been described earlier, the depiction has been mainly qualitative in nature [13], which may lead to the underappreciation of the small size of the capsule. The arches inserted above the radial and coronoid fossae at a mean length of 13.5 mm and 11.6 mm, respectively, above the TEL in a plane perpendicular to the TEL. The nadir of the synovial membrane inserted just above the lateral end of the trochlea 3.6 mm above the TEL (Table 4). These results demonstrate that the joint capsule attachments of the synovial membrane are smaller above the coronoid and radial fossae than the broad fibrous capsular attachments (Figure 1). As a result, the interspace between the synovial membrane and the outer fibrous capsule may prove to be a confusing region for novice surgeons performing elbow arthroscopy, primarily because the outer fibrous capsule may be penetrated without necessarily entering the joint space. This may be the case if the scope becomes lodged in the interspace between the two layers of the capsule.

Following trauma, the structure of the anterior capsule is altered leading to pathologic thickening and disorganization of the collagen fiber arrangement as well as involvement of cytokines leading to the final outcome of elbow contracture and stiffness [3]. The nadir of the SM between the two fossae may help to explain a reduction in working arthroscopic volume in inflammatory and posttraumatic pathologies because the spanning distance of the anterior capsule between the humerus and ulna appears to be the shortest at this point.

There were no significant side specific differences amongst our specimens. The major differences observed were gender related and this was expected, as the width of the distal humerus (as defined by the TEL) was greater in males. The SM was on average only 6.5 mm medial to the lateral epicondyle apex. Highlighting this close proximity may be of benefit for physicians attempting to inject inflamed extensor tendon origins in this territory and may help avoid penetrating the lateral capsule with a resultant intra-articular injection. This close proximity may also be helpful if planning to arthroscopically treat lateral epicondylitis [14].

The synovial membrane was continuous and encapsulated the entire anterior and posterior articular surfaces of the distal humerus (Figure 4). The axis of the synovial membrane of the capsule was clearly oriented in valgus relative to the axis of the distal humerus in 81.8% of cases (Figure 6), which coincided with the flexion-extension axis of the ulnohumeral joint. In general, the flexion-extension axis of the elbow is in 4–8° of valgus relative to the long axis of the humerus [15, 16]. Interestingly, in 18 out of 22 specimens (81.8%), an infolding extension of the SM was observed overlying the medial aspect of the trochlea (Figures 7(a) and 7(b)). The texture of this segment of the capsule was thick and ligamentous and at times resembling fibrocartilage, but no histological study was performed, which is a goal for future studies. This observation leads us to believe that the medial part of the anterior capsule forms an articular surface of the elbow joint and may be involved in load absorption, potentially acting as a shock absorber. This concept is similar to capsular interposition between the joint surfaces in the articular discs of the knees, acromioclavicular joint, sternoclavicular joint, or the interphalangeal joints of the fingers or toes, where part of the capsule is used as a bearing surface [17].

One of the limitations of this study is the small number of specimens studied. However, a unique method of data collection and analysis was utilized, which is our starting point for future clinically relevant morphometric mapping. In addition, despite the small numbers, the qualitative structural patterns were observed in the overwhelming majority of specimens (Table 6). Also, we only studied the footprints and dimensions of the synovial membrane attachments of the elbow capsule, while neglecting the loose, fibrous external capsule. Information regarding the inner synovial capsule however is relevant particularly in regard to arthroscopic access and navigation of the elbow joint.

The strengths of this study lie in the methodology of data collection and analysis. Three-dimensional anatomical studies can provide more information than traditional two-dimensional studies and enabled reproducible and accurate soft tissue mapping and analysis.

## 5. Conclusion

In conclusion, the humeral footprint of the synovial membrane of the anterior elbow capsule was quantitatively and qualitatively studied in considerable three-dimensional and two-dimensional detail. We found that the synovial membrane has a distinctive double arched shape overlying the radial and coronoid fossae. Part of the anterior capsule folds into the medial joint space over the trochlea, with an indeterminate function. Objective normalized measurements of the anatomic landmarks and of the synovial membrane may be of benefit for surgeons and health care providers treating elbow disease. This level of anatomical knowledge can assist surgeons when trying to create working space during arthroscopic procedures but can also help in understanding that detachment of the synovial footprint does not equate to releasing the outer fibrous capsule, in joint contracture release procedures.

## Figures and Tables

**Table 1:** Qualitative description of the anterior capsule.

Structure	Structure present, *n* = 22
Double arched capsular insertion	22 (100%)

Nadir of anterior capsule insertion overlying the lateral end of trochlea	22 (100%)

Medial infolding of capsule overlying the medial trochlea	18 (81.8%)

Axis of capsular insertion rotated medially	18 (81.8%)

Capsular insertion on flat bony convexity above the depression of the coronoid fossa	18 (81.8%)

Capsular insertion on flat bony segment above the depression of the radial fossa	21 (95.5%)

**Table 2:** The dimensions of the transepicondylar line (TEL).

	Mean	Median	SD	Range	*P* value
Length of TEL (mm)					
Male (*n* = 12)	70.9	69.8	4.1	65.8–81.5	0.000
Female (*n* = 10)	61.9	61.9	3.3	57.9–67.4	
					
Right (*n* = 11)	67.4	67.4	6.4	59.0–81.5	0.748
Left (*n* = 11)	66.1	66.1	5.6	57.9–76.6	
					
Total	66.8	67.3	5.9	57.9–81.5	

TEL: transepicondylar line.

**Table 3:** Landmarks of the anterior humeral capsule.

	Mean	Median	SD	Range	*P* value
Distance from ME to the apex of the capsule overlying the coronoid fossa along the TEL (mm)					
Male	32.5	33.4	2.8	28.5–37.0	0.043
Female	29.8	29.7	1.7	27.7–33.7	
					
Right	32.3	33.4	3.0	27.7–37.0	0.088
Left	30.3	29.6	2.0	28.0–34.5	
					
Total	31.3	30.1	2.7	27.7–37.0	

Proportion of TEL length from the ME to the apex of the capsule overlying the coronoid fossa (%)					
Male	45.9	45.2	2.8	42.0–50.6	0.069
Female	48.2	48.7	2.3	44.9–50.9	
					
Right	48.0	48.7	2.4	43.7–50.6	0.116
Left	45.9	45.0	3.0	42.0–50.9	
					
Total	46.9	47.4	2.9	42.0–50.9	

Distance from the ME to the capsular nadir along the TEL (mm)					
Male	43.3	43.1	2.6	38.8–48.4	0.000
Female	37.1	37.3	2.3	34.3–40.4	
					
Right	40.8	40.4	4.4	34.3–48.4	0.898
Left	40.2	41.6	3.7	34.7–46.4	
					
Total	40.5	41.0	4.0	34.3–48.4	

Proportion of TEL length from the ME to capsular nadir (%)					
Male	61.2	60.9	2.4	57.7–64.7	0.456
Female	60.0	60.4	2.5	56.0–62.6	
					
Right	60.5	59.9	2.8	56.0–64.7	0.748
Left	60.9	60.6	2.1	56.0–64.3	
					
Total	60.7	60.6	2.4	56.0–64.7	

Distance from ME to the apex of the capsule overlying the radial fossa along the TEL (mm)					
Male	47.4	47.8	3.6	39.1–52.5	0.002
Female	41.9	41.5	3.3	37.8–47.6	
					
Right	45.7	47.1	4.0	38.6–51.2	0.401
Left	44.1	44.5	4.8	37.8–52.5	
					
Total	44.9	45.0	4.4	37.8–52.5	

Proportion of TEL from ME to Apex of radial fossa (%)					
Male	66.9	67.3	3.7	59.4–71.9	0.722
Female	67.7	67.9	2.6	63.1–71.8	
					
Right	67.9	67.9	2.6	62.8–71.4	0.365
Left	66.6	67.2	3.7	59.4–71.9	
					
Total	67.3	67.9	3.2	59.4–71.9	

TEL: transepicondylar line; ME: medial epicondyle; LE: lateral epicondyle.

**Table 4:** Vertical landmarks of the anterior humeral capsule.

	Mean	Median	SD	Range	*P* value
Vertical distance from the TEL to the capsular apex overlying the coronoid fossa (mm)					
Male (*n* = 12)	12.3	12.9	3.4	6.3–17.9	0.254
Female (*n* = 10)	10.8	10.8	2.8	4.8–15.3	
					
Right (*n* = 11)	11.6	12.7	3.6	4.8–17.9	0.562
Left (*n* = 11)	11.6	11.3	2.8	7.5–17.0	
					
Total	11.6	12.2	3.2	4.8–17.9	

Vertical distance from the TEL to the capsular apex overlying the radial fossa (mm)					
Male	13.8	13.1	3.4	8.6–19.6	0.674
Female	13.0	13.2	3.3	7.6–18.2	
					
Right	13.7	12.9	3.4	7.6–18.5	0.748
Left	13.3	13.3	3.3	8.6–19.6	
					
Total	13.5	13.1	3.3	7.6–19.6	

Vertical distance from the TEL to the capsular nadir overlying the lateral edge of the trochlea (mm)					
Male	3.5	3.1	2.1	0.5–9.6	0.771
Female	3.8	3.8	3.9	−2.2–10.2	
					
Right	4.00	3.4	3.2	−2.2–9.6	0.332
Left	3.3	2.7	2.8	−1.9–10.2	
					
Total	3.6	3.1	3.0	−2.2–10	

TEL: transepicondylar line.

**Table 5:** The edges of the internal capsular insertion.

	Mean	Median	SD	Range	*P* value
Distance from ME to medial edge of capsule along TEL (mm)					
Male	23.8	23.7	3.8	18.3–31.7	0.771
Female	22.5	23.6	4.5	16.0–28.0	
					
Right	23.6	23.4	4.5	16.0–31.7	0.652
Left	22.8	23.9	3.8	16.0–28.0	
					
Total	23.2	23.7	4.1	16.0–31.7	

Proportion of TEL from ME to capsule along TEL (%)					
Male	33.6	34.2	4.2	26.8–38.9	0.381
Female	36.4	37.7	7.3	27.1–47.6	
					
Right	35.1	36.4	6	27.1–44.2	0.699
Left	34.6	34.2	5.9	26.8–47.6	
					
Total	34.8	34.5	5.8	26.8–47.6	

LE to Lateral edge of capsule along TEL (mm)					
Male	6.7	6.5	3.7	1.2–12.0	0.872
Female	6.4	6.3	2.2	3.6–10.6	
					
Right	6.9	6.9	3.4	1.2–12.0	0.699
Left	6.2	6.1	2.8	1.9–11.0	
					
Total	6.5	6.3	3.1	1.2–12.0	

Proportion of TEL from LE to lateral edge of capsule along TEL (%)					
Male	9.3	9.2	5.2	1.8–17.5	0.628
Female	10.3	10.1	3.4	6.0–16.7	
					
Right	10.1	9.6	4.9	1.8–17.5	0.797
Left	9.4	9.8	4	2.9–15.8	
					
Total	9.8	9.7	4.4	1.8–17.5	

TEL: transepicondylar line; ME: medial epicondyle; LE: lateral epicondyle.

**Table 6:** Distal humerus surface area encompassed by the capsule.

	Mean	Median	SD	Range	*P* value
Surface area encompassed by inner capsule (cm^2^)					
Male (*n* = 12)	42.2	42.3	3.6	36.4–48.0	0.000
Female (*n* = 10)	33.6	33.8	4.1	26.5–39.8	
					
Right (*n* = 11)	38.6	39.8	6.0	27.6–48.0	0.797
Left (*n* = 11)	38.0	37.8	5.7	26.5–45.9	
					
Total	38.3	38.7	5.8	26.6–48.0	

## References

[1] Johnson D., Tytherleigh-Strong G., Standring S. (2008). Elbow. *Gray's Anatomy*.

[2] Morrey B. F., An K. N. (1983). Articular and ligamentous contributions to the stability of the elbow joint. *The American Journal of Sports Medicine*.

[3] Safran M. R., Baillargeon D. (2005). Soft-tissue stabilizers of the elbow. *Journal of Shoulder and Elbow Surgery*.

[4] Hsu J. W., Gould J. L., Fonseca-Sabune H., Hausman M. H. (2009). The emerging role of elbow arthroscopy in chronic use injuries and fracture care. *Hand Clinics*.

[5] Kelly E. W., Morrey B. F., O'Driscoll S. W. (2001). Complications of elbow arthroscopy. *The Journal of Bone & Joint Surgery—American Volume*.

[6] Thoreux P., Blondeau C., Durand S., Masquelet A. C. (2006). Anatomical basis of arthroscopic capsulotomy for elbow stiffness. *Surgical and Radiologic Anatomy*.

[7] Bruno R. J., Lee M. L., Strauch R. J., Rosenwasser M. P. (2002). Posttraumatic elbow stiffness: evaluation and management. *The Journal of the American Academy of Orthopaedic Surgeons*.

[8] Cohen M. S., Schimmel D. R., Masuda K., Hastings H., Muehleman C. (2007). Structural and biochemical evaluation of the elbow capsule after trauma. *Journal of Shoulder and Elbow Surgery*.

[9] Hildebrand K. A., Zhang M., Hart D. A. (2005). High rate of joint capsule matrix turnover in chronic human elbow contractures. *Clinical Orthopaedics and Related Research*.

[10] Gallay S. H., Richards R. R., O'Driscoll S. W. (1993). Intraarticular capacity and compliance of stiff and normal elbows. *Arthroscopy*.

[11] Miller R., Dlabach J., Canale S. T., Beaty J. H. (2007). Elbow and shoulder injuries. *Campbell's Operative Orthopaedics*.

[12] Stothers K., Day B., Regan W. R. (1995). Arthroscopy of the elbow: anatomy, portal sites, and a description of the proximal lateral portal. *Arthroscopy: The Journal of Arthroscopic & Related Surgery*.

[13] Moore K. L. (1992). *Clinically Oriented Anatomy*.

[14] Cohen M. S., Romeo A. A., Hennigan S. P., Gordon M. (2008). Lateral epicondylitis: anatomic relationships of the extensor tendon origins and implications for arthroscopic treatment. *Journal of Shoulder and Elbow Surgery*.

[15] Johnson J. A., King G. J., Williams G. R., Yamaguchi K., Ramsey M. L. (2005). Anatomy and biomechanics of the elbow. *Shoulder and Elbow Arthroplasty*.

[16] Johnson J. A., Rath D. A., Dunning C. E., Roth S. E., King G. J. W. (2000). Simulation of elbow and forearm motion in vitro using a load controlled testing apparatus. *Journal of Biomechanics*.

[17] Ralphs J. R., Benjamin M. (1994). The joint capsule: structure, composition, ageing and disease. *Journal of Anatomy*.

